# Detection and extraction of heavy metal ions using paper-based analytical devices fabricated via atom stamp printing

**DOI:** 10.1038/s41378-019-0123-9

**Published:** 2020-03-09

**Authors:** Yanfang Guan, Baichuan Sun

**Affiliations:** School of Electromechanical Engineering, Henan University of technology, Zhengzhou, 450001 China

**Keywords:** Chemistry, Electrical and electronic engineering

## Abstract

As a promising concept, microfluidic paper-based analytical devices (μPADs) have seen rapid development in recent years. In this study, a new method of fabricating μPADs by atom stamp printing (ASP) is proposed and studied. The advantages of this new method compared to other methods include its low cost, ease of operation, high production efficiency, and high resolution (the minimum widths of the hydrophilic channels and hydrophobic barriers are 328 and 312 μm, respectively). As a proof of concept, μPADs fabricated with the ASP method were used to detect different concentrations of Cu^2+^ via a colorimetric method. Moreover, combined with a distance-based detection method, these devices achieved a Cu^2+^ detection limit of down to 1 mg/L. In addition, a new paper-based solid–liquid extraction device (PSED) based on a three-dimensional (3D) μPAD with a “3 + 2” structure and a recyclable extraction mode was developed. Specifically, using the characteristics of paper filtration and capillary force, the device completed multiple extraction and filtration steps from traditional solid–liquid extraction processes with high efficiency. The developed PSED platform allows the detection of heavy metal ions much more cheaply and simply and with a faster response time at the point of care, and it has great promise for applications in food safety and environmental pollution in resource-limited areas.

## Microfluidics: Putting a stamp on extraction

Researchers in China have developed a method of making microfluidic paper-based analytical devices (µPADs) and used it to fabricate a µPAD which detects and extracts heavy metal ions. A team led by Yanfang Guan and Baichuan Sun of Henan University of Technology tested µPAD fabrication via atom stamp printing, in which laser-engraved stamps are used to print hydrophobic channels on paper. The technique produced µPADs with channels around 300 µm wide at a relatively low cost. Next, the team used this approach to create a 3D µPAD which could detect and extract Cu, Zn, Pb. etc. from a solution. The device preformed at least as well as traditional methods but is portable, cheaper, and easier to use. This proof of concept demonstrates the value and utility of such devices in areas such as food safety and pollution management.

## Abstract (web summary)

In this paper, we propose a new method of making *μ*PADs: atomic stamp printing (ASP). At the same time, we have made a new device for extracting heavy metal ions from soil: PSED, which is more economical and convenient while satisfying the extraction effect.

## Graphical abstract

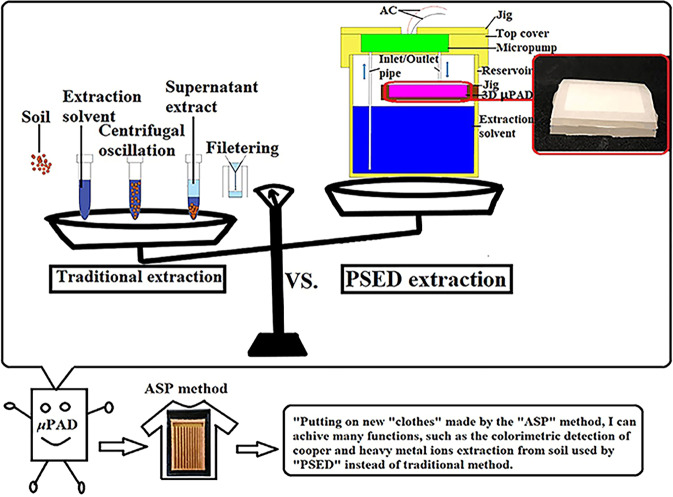

## Introduction

In the 1990s, the concept of a “lab on a chip (LOC)” was proposed based on the development of microfluidic technology^1,2^. The main feature of this technology is the ability to perform biochemical analysis on a chip that is a few square centimeters or smaller and to realize sample mixing, purification, separation, and other operations, thus saving on both space and cost. Microfluidic paper-based analytical devices (μPADs) were first proposed by Martinez et al.^3^ as a device to replace traditional microfluidic chips, such as chips based on glass^4,5^ and silicon^6,7^, with the advantages of simple fabrication, low cost, portability, and disposability, and they have widespread applications in the point-of-care testing (POCT) field^8^.

The principle of fabricating μPADs is to hydrophobically treat paper substrates to form distinct hydrophobic and hydrophilic areas, thus restricting and guiding fluid flow. At present, there are a number of methods that can be used to fabricate μPADs, such as photolithography^3,9^, wax printing^10–14^, paper cutting^15^, drawing^16^, inkjet printing^17–19^, laser cutting^20^, and stamping^21–26^. For the first time, Martinez et al.^3^ used photolithography technology with a SU-8 photoresist to fabricate a hydrophobic pattern on paper. He et al.^9^ fabricated μPADs with high resolution (hydrophilic channel was 233 ± 30 μm wide) using filter paper that was preprocessed with octadecyl trichlorosilane solution and deep ultraviolet light.

To achieve rapid and inexpensive production, Lu et al.^10,11^ and Carrilho et al.^12^ produced μPADs by wax printing. The process of patterning paper with wax is quite simple, and only a commercial wax printing machine and a heating board or oven are needed in device production, which can be conducted at home. If μPADs with high resolution are not needed in the experiment, simple paper cutting^15^ and drawing^16^ can be used for their production, but the production efficiency is lower than that of other methods. In 2008, Abe et al.^17^ completed the fabrication of μPADs by inkjet printing with “chemical sensing inks” and achieved a relatively high channel accuracy of 550 μm. Up to now, laser cutting has been the best way to achieve the highest accuracy of channel resolution on paper, which can reach 62 ± 1 μm and can be used on any paper with a hydrophobic surface coating^20^, such as wax paper or parchment paper; however, this operation is more complex, and the paper needs further chemical treatment.

Stamping, as another production technology, has been widely used, and different types of stamps can be used, such as a stamp with paper and tape^21^, a polydimethylsiloxane (PDMS) high-relief stamp^22^, an iron^23^ or steel stamp^24^, and a flash foam stamp (FFS)^25,26^. The hydrophobic material is printed on paper by the stamps, which are designed to be the desired pattern. Most of the above stamps are hard stamps, which are difficult to make and have the same disadvantages as wax printing, such as low resolution, because wax is commonly used as the hydrophobic solvent. An FFS overcomes these shortcomings, realizing a relatively high resolution using PDMS as the hydrophobic solvent and has the advantages of simple operation and low cost^25^. However, it takes a long time to prepare, especially when the stamps are immersed in PDMS solvent, which takes more than half an hour for complete production. In addition, the stamp can only be printed several times after each soaking and must be resoaked, which takes a certain amount of time. Therefore, when considering the type of stamp to use, it is necessary to consider the cost of time while utilizing the economic benefits and convenience.

Atom stamps (ASs), also called machine-engraved penetrating stamps, can be manually engraved or can be made by a laser engraving machine and have the advantages of low cost, high efficiency, and high resolution. In situations where high resolution is not needed for μPADs, it is possible to carve the stamp pad manually. While manual production saves on cost, it requires practice and skill. A laser engraving machine works with common drawing software, such as AutoCAD and CorelDRAW, to create pattern designs; therefore, an AS is also known as a laser engraving seal stamp. Printing can be performed with an AS because the stamps can absorb ink due to their microporous structure, and the process is simple. In this study, an AS was used as a new method to produce μPADs, termed ASP (AS printing). For μPAD production, a stamp of the required pattern needs to be soaked in PDMS solvent, then printed on paper, and left in a vacuum drying box or oven for a moment to complete the entire fabrication process.

To date, there have been many methods to detect heavy metal ions based on μPADs^27^, such as colorimetric detection^28–30^, fluorescence detection^31^, and electrochemical detection^32^. Xu et al.^28^ simultaneously detected iron and nickel ions by a colorimetric method through “multichannel” μPADs, which proved the effectiveness of μPADs in detecting heavy metal ions. Fu et al.^31^ made a new paper-based sensor using the fluorescence quenching function of gold nanoparticles (Au NPs), and the detection procedure was completed in 15 min. Hu et al.^32^ used paper-based capacitive sensors (PCSs) to realize chemical identification and quantitative detection, including the quantitative detection of heavy metal ions; this approach integrated electrochemical methods and paper-based methods to eliminate the need for large-scale equipment in the detection procedure, making it more environmentally friendly. Among these three methods, the colorimetric method is the most commonly used because it is simple, is easy to operate, and does not require complex equipment to meet the basic needs of on-site detection. However, it is difficult to achieve accurate quantitative detection with the colorimetric method, and there is low accuracy with this method. Electrochemical detection overcomes the problem of low accuracy and can achieve quantitative detection, but it is relatively difficult to perform and requires relatively expensive testing equipment. Fluorescence detection can achieve targeted detection with high accuracy and reduce some necessary interference; however, it needs special detection instruments or reagents, and it is challenging to use. In this study, we chose the colorimetric method to detect Cu^2+^.

As a proof of concept, μPADs fabricated via ASP were used to perform semiquantitative detection of Cu^2+^ by a colorimetric method^33^. Alternatively, distance-based detection can be used for detection instead of the colorimetric method^34–36^, and it is an effective quantitative method with better accuracy. The distance-based method was first proposed by Cate et al.^35^, and they demonstrated the method on μPADs for the detection of glucose, nickel, and so on. Later, Pratiwi et al.^36^ used a porphyrin derivative to detect copper ions using the distance-based method, and the minimum detection limit achieved was 1 mg/L. In this study, we combine the colorimetric method with the distance-based method to achieve quantitative detection of the Cu^2+^ concentration.

To further demonstrate the versatility of our μPADs, we introduced an integrated device, which combined 3D μPADs and a homemade micropump to achieve integrated extraction and filtration, called a paper-based soil–liquid extraction device (PSED). Usually, the solid–liquid extraction process of heavy metal ions in soil includes centrifugation, oscillation, and more^37^, which require specialized equipment such as a centrifuge^38^ and ultrasonic vibration^39^. Therefore, the traditional process does not match the principles of low cost, portability, and miniaturization in the POCT field^40^, and it is necessary to develop a cheap and simple extraction device to meet the needs of on-site detection in resource-limited areas. The PSED studied here capitalizes on the advantages of paper itself, such as its low cost, portability, and filterability, and demonstrated superior performance when used as an extraction device in our experiment.

## Results and discussion

### Resolution analysis of μPADs by the ASP method

**Fig. 1:**
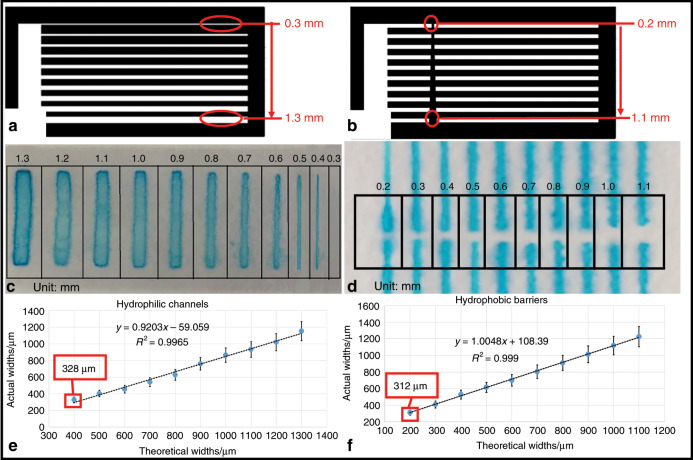
Resolution testing of μPADs made by the ASP method. **a** Structural picture of the hydrophilic channels. **b** Structural picture of the hydrophobic barriers. **c** Hydrophilic channels tested with blue dye. **d** Hydrophobic barriers tested with blue dye. **e** Comparison of the theoretical and actual widths of the hydrophilic channels. **f** Comparison of the theoretical and actual widths of the hydrophobic barriers.

Resolution is an important metric to evaluate the performance of μPADs. Resolution refers to the minimum channel width of the fluid flow passages on the paper and the minimum width of the hydrophobic barrier that prevents the flow of fluid. The structure of the hydrophilic channels (0.3–1.3 mm) and hydrophobic barriers (0.2–1.1 mm) was designed as shown in Fig. 1a, b. To observe the flow through the hydrophilic channels and the hydrophobic barriers accurately, a blue dye was added to the channels. As shown in Fig. 1c, d, it was obvious that when the width of the hydrophilic channel was 0.4 mm, the liquid could flow normally, and when the hydrophobic barrier was 0.2 mm, the flow was blocked. As seen in Fig. 1e, f, the theoretical widths of the hydrophilic channels and hydrophobic barriers are different from their actual widths in the μPADs.

The actual widths of the hydrophilic channel were generally smaller than the theoretical widths; on the contrary, the actual widths were generally larger than the theoretical widths for the hydrophobic barriers according to the measurement results and the fitting line. This phenomenon is due to the diffusion and permeation of PDMS that occurred during the printing of the hydrophobic materials on the filter paper by AS fabrication. Furthermore, there is an approximately proportional relationship between the actual value and the theoretical value, and the correlation coefficient (*R*^2^) is ~0.9965 for the hydrophilic channels and 0.999 for the hydrophobic barriers. The actual minimum widths of the hydrophilic channels and hydrophobic barriers measured under a microscope were 328 and 312 μm (Fig. 1e, f), respectively.

Among μPADs made by different methods, μPADs made by laser cutting can achieve the highest resolution, with a minimum hydrophilic channel width of 62 ± 1 μm^20^. However, special paper materials are needed, the cost is high, and the operation is complex. Drawing and cutting have low costs and are easy to operate, while the resolution (the width of the hydrophilic channels was ~2000 μm) is low^15,16^. Wax printing (600 μm)^﻿12﻿^ and inkjet printing (550 μm)^17^ require special printers. Wax printers are seldom sold on the market, and inkjet printing requires customized printers. FFS lithography (FFSL) and ASP are stamping methods, but the resolution of the FFSL method (the widths of hydrophilic channels and hydrophobic barriers were 632 ± 27 and 306 ± 20 μm, respectively)^25^ is significantly lower than that of the ASP method. Additionally, ASP is more efficient than FFSL in the production of μPADs. A detailed comparison of the μPAD fabrication methods is shown in Table 1.

**Table 1:** Comparison of the μPAD fabrication methods.

Method	Channel (μm)	Barrier (μm)	Advantages	Disadvantages
Photolithography^3,9^	233 ± 30	137 ± 21	High resolution	Complex operation; high cost
Wax printing^12^	~600	~1300	Easy to fabricate, low cost	Low resolution
Inkjet printing^17^	550	302	High resolution, rapid	Special printers
Cutting^15^	~2000	–	Low cost, easy to operate	Low resolution
Laser cutting^20^	62 ± 1	80	High resolution	Complex operation, special paper materials are needed
FFSL^25^	632 ± 27	306 ± 20	Low cost, easy to fabricate	Low resolution
ASP	328	312	Low cost, rapid, high resolution, easy to fabricate	

### Economic analysis of the ASP method

As mentioned, the advantages of the ASP method include its low cost and easy operation. The most expensive one-time cost is the laser engraving machine. Nonetheless, the laser engraving machine is highly efficient, producing ~20 stamps in a min, and is manufactured anywhere that produces seals. The total cost of the other materials is ~9 cents (¥0.59/$0.09), as seen in Table 2, which is lower than the cost of the FFSL method^25^ (¥0.91/$0.15). Each AS can be reused many times, which greatly reduces the cost of production.

**Table 2:** Cost comparison of the ASP and FFSL methods.

Materials	Amount	Cost of FFSL	Cost of ASP
Filter paper	40 × 40 mm^2^	¥0.15	¥0.15
Atom stamp	40 × 40 mm^2^	–	¥0.03
PDMS	0.5 g	¥0.4	¥0.4
Flash foam	40 × 40 mm^2^	¥0.09	–
Tracing paper	40 × 40 mm^2^	¥0.001	–
Mask	1 piece of paper	¥0.01	–
Electricity	≈0.1 kw/h	¥0.01	¥0.01
Total		¥0.91/$0.15	¥0.59/$0.09

### Colorimetric analysis of Cu^2+^

#### Colorimetric card detection of Cu^2+^

**Fig. 2:**
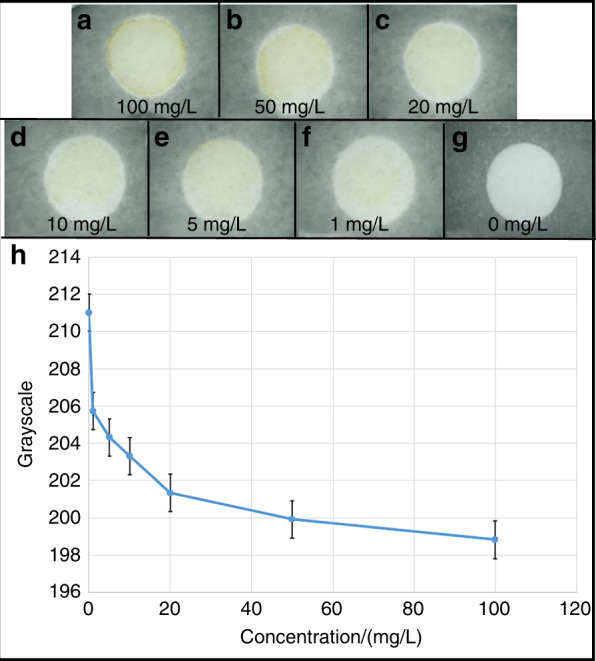
Pictures of the colorimetric detection of Cu^2+^. **a**–**g** Color rendering effect of copper ions and DDTC at different concentrations. **h** Grayscale trend with the concentration of Cu^2+^.

As shown in Fig. 2a–g, the μPADs with Cu^2+^ clearly change from white to yellow, and the color of the yellow complexes deepens with increasing Cu^2+^ concentration. The image was processed by the ImageJ software, as shown in Fig. 2h, and with an increase in ion concentration, the grayscale value decreased (gray value of 255 represents white or no color, and gray value of 0 represents black or darker color), indicating that the color of the complex deepened.

#### Distance-based detection of Cu^2+^

**Fig. 3:**
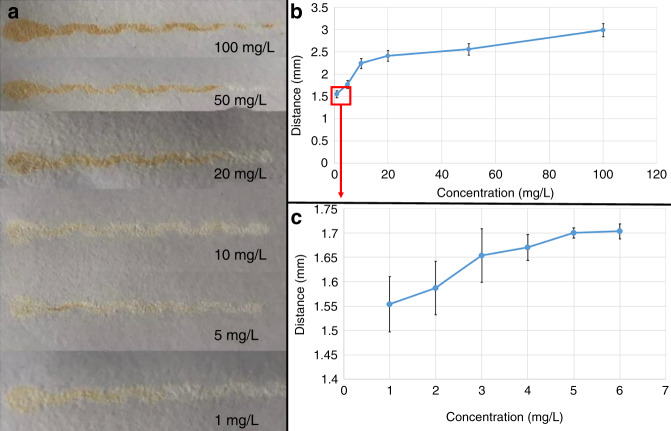
Testing of the Cu^2+^ concentration based on the distance-based detection method. **a** The flow of the solution on the channel with increasing Cu^2+^ concentration. **b** Linear relationship between Cu^2+^ concentration (0–100 mg/L) and flow distance in the channel. **c** Flow length at 1–6 mg/L Cu^2+^ concentrations.

Figure 3a shows the distances of Cu^2+^ solutions with different concentrations flowing through the channels of the μPADs. The length of the yellow-colored band increased as the concentration of Cu^2+^ increased. As shown in Fig. 3b, c, the colored band length was constant above 100 mg/L, making it the upper limit of the device. The minimum detection concentration of Cu^2+^ was 1 mg/L, in accordance with the World Health Organization and United States Environmental Protection Agency regulations for the maximum contamination concentration of Cu^2+^ in drinking water, which are 2 and 1.3 mg/L^41,42^, respectively.

### Heavy metal ion extraction in soil

**Fig. 4:**
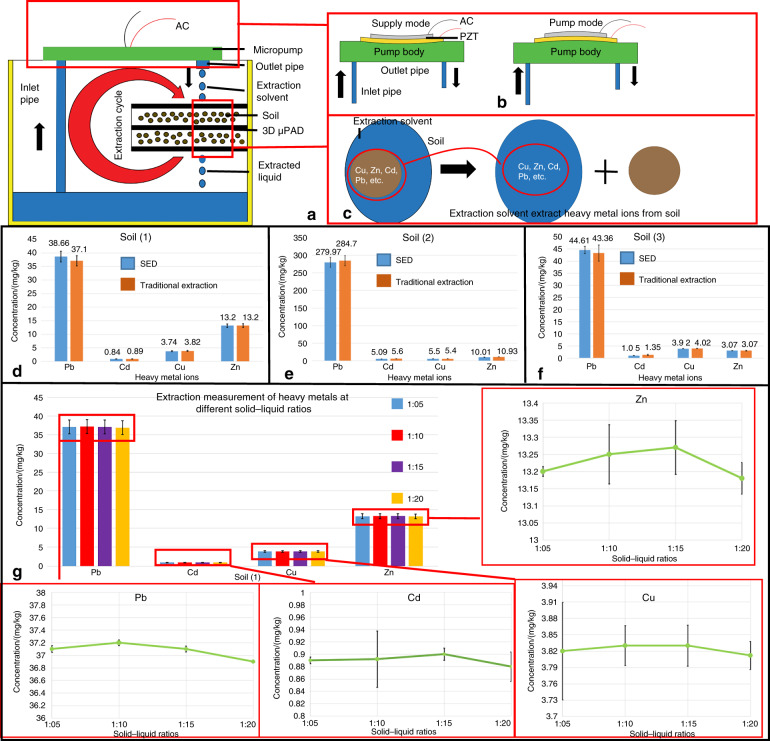
Working principle and comparison of detected heavy metal ion concentrations between the PSED and traditional extraction method. **a**–**c** Schematic of the working principle of the PSED. **d**–**f** The concentration results of the heavy metal ions in soils (1), (2), and (3), respectively. **g** Comparison of the heavy metal ion concentrations obtained by PSED extraction at different solid–liquid ratios.

The working principles of a PSED are shown in Fig. 4a–c. Two main steps occur during extraction: the micropump driving^43^ step, which enables the device to achieve the extraction cycle shown in Fig. 4b, and the extraction step by the 3D μPAD, which utilizes the microporous characteristics of the filter paper itself to complete the solid–liquid extraction and filtration processes, as shown in Fig. 4a, c. First, the soil samples were stored on top of the 3D μPAD. Then, the extraction solvent flowed out from the outlet pipe of the micropump, mixed with the soil, and flushed down into the reservoir. Concomitantly, the extraction solvent solubilized the heavy metal ions, sucked them through the inlet pipe, and was pumped out again, forming the extraction cycle. Finally, the heavy metal ions, such as Cu, Zn, Cd, and Pb, were extracted from the soil samples via the continuous supply and pump cycle of the micropump.

As seen from Fig. 4d–f, the concentration of the heavy metal ions (Pb, Cd, Cu, Zn, etc.) obtained by the PSED extraction procedure was almost the same as that obtained by traditional methods, which proves that the PSED was effective. The error rate between the PSED extraction and traditional extraction process was between 0.02 and 0.05, which is acceptable in extraction process (the error rate = |heavy metal ion concentration obtained by PSED extraction–heavy metal ion concentration obtained by traditional extraction|/heavy metal ion concentration obtained by traditional extraction). When the error rate is <0.05, a method is qualified for extraction^44^. In the experiment, solid–liquid ratios of 1:5, 1:10, 1:15, and 1:20 were adopted for the extraction process (Fig. 4g), and under the condition of satisfying the extraction accuracy, the solid–liquid ratio of 1:10 completed the detection. By contrast, the error rates of the ratios of 1:15 and 1:20 (0.06 and 0.09, respectively) were larger than that of the ratio of 1:10, and therefore, these ratios were not recommended for use in extractions.

The extractant volume should not be too small for the extraction process. First, there will be losses of the extractant during the extraction process, which will remain in the μPADs, soil, or devices. Second, if there is too little final extraction solvent, detection will be difficult. Therefore, more than 30 mL of the extractant is required. This experiment used 40 mL of extractant and 4 g of soil, and the solid–liquid ratio was 1:10. To ensure complete extraction, we also compared the extraction time and found that 20 min was sufficient for complete extraction of the heavy metal ions. Each 3D μPAD can hold 2 g of soil, and therefore, the extraction procedure takes 40 min to complete.

Compared with the traditional extraction method, 3D μPAD extraction omitted the process of filtration, which makes the operation simpler and the extraction accuracy higher. Moreover, 3D μPADs have the advantages of being portable and cheap, and removing the need for large equipment, which makes extraction simple. It is worth noting that the size of the device can be adjusted according to different needs, providing more flexibility for real-world applications.

## Conclusions

μPADs, as a new type of detection device, have been used in many fields, such as environmental monitoring and food safety. The fabrication technology of μPADs has become a focus of research in the past few years. In this study, a new μPAD fabrication method using ASP was proposed. The μPADs fabricated with ASP achieved high resolution, 328 μm for the hydrophilic channels and 312 μm for the hydrophobic barriers. Compared with other methods, ASP is low cost and has simple operation, short preparation time, high resolution, and higher sensitivity than other methods (wax printing, inkjet printing, photolithography method, etc.). Moreover, this method can be used in most situation because PDMS is a transparent liquid, avoiding the possible contamination of the hydrophobic channels by the hydrophobic agent. We demonstrated that ASP-fabricated μPADs can detect Cu^2+^ using a colorimetric method and that, combined with a distance-based detection method, the approach can achieve the detection of Cu^2+^ at a concentration of 1 mg/L.

In addition, a new solid–liquid extraction device, PSED, was proposed to extract heavy metal ions from the soil. This device integrated the extraction, filtration, and collection processes and requires fewer experimental samples to meet the needs of POCT by reducing sample loss. In addition, the whole device has high extraction efficiency, low cost, and no pollution, and it can be improved according to its own requirements and can meet the demands of most solid–liquid extraction processes. This device is also not limited to soil. Moreover, the device is simple to manufacture and can be produced with low cost (3D printing). Therefore, it is suitable for use in areas with no professional equipment and low income and can be adjusted according to extraction needs. However, the simple operation, highly integrated nature, and production of real POCT products need to be improved for use of this device.

## Materials and methods

### Materials and devices

Whatman No. 1, No. 4, and No. 5 filter papers were purchased from Whatman Company, UK. PDMS (Sylgard 184) was purchased from Dow Corning, USA. Standard copper solution (1.0 g/L) was purchased from National Standard Substances Research Center, China. Ammonia and copper reagents were purchased from Brilliant, China. DTPA (diethylenetriamine pentaacetic acid) extraction solution was made in the Chemistry Research Laboratory of Henan University of Technology. Deionized water was homemade. Soil samples were collected from three different areas: No. 1 (Lingshan Village of Jiyuan, China), No. 2 (smelting plant in Zhengzhou, China), and No. 3 (Tangshi greenhouse, China). The laser engraving machine was purchased from Laser Star, China. The atomic absorption spectrometer was purchased from Thermo Fisher, USA. The fused deposition molding (FDM) 3D printer (D-Force 400) was purchased from Triangle Laboratory Co., Ltd. (Jiangsu, China). The power amplifier and the micropump were homemade.

### Paper selection

The first step to make μPADs is choosing the right type of paper. In this experiment, three types of filter paper were selected for comparison: Whatman No. 1, No. 4, and No. 5 filter papers. Among them, No. 1 filter paper has the advantages of moderate pore size and moderate flow rate and is easy to observe and detect; therefore, it is often used to make μPADs^45^. No. 4 filter paper, because of its large aperture, has a faster flow rate on the paper, and it is not easy to observe the fluid’s flow and reaction. Moreover, it is thicker than No. 1 filter paper, making it difficult for the hydrophobic agent to penetrate its interior. Fluid flows slowly on No. 5 filter paper since the pore size is small, which is also not easy for hydrophobic solvents to penetrate and makes it hard to perform paper bonding. Therefore, No. 1 filter paper was employed as the paper base in the experiment and production process. The papers’ parameters are listed in Table 3.

**Table 3:** Performance parameters of the different types of filter paper.

Type	Aperture (μm)	Filtration speed	Thickness (μm)	Basic weight (g/m^2^)
No. 1	11	Medium	180	88
No. 4	20–25	Fast	205	96
No. 5	2.5	Slow	200	105

### Fabrication of the AS

**Fig. 5:**
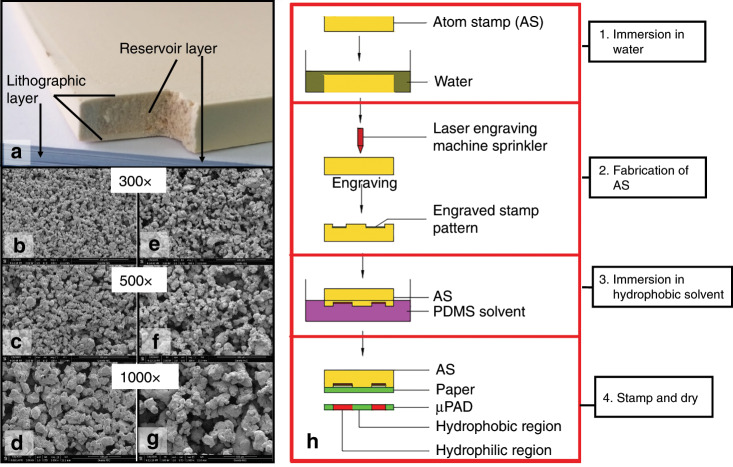
Internal composition and fabrication progress of the AS. **a** Components of the atom stamp. **b**–**d** Microstructure of the lithographic layer. **e**–**g** Microstructure of the reservoir layer. **h** Fabrication process of an ASP.

The AS is made of a special sponge material with a microporous structure, which is divided into a reservoir layer (storing ink) and a lithographic layer (engraving pattern), as shown in Fig. 5a. The different structures of the reservoir layer and lithographic layer are shown in Fig. 5b–g, respectively. As shown in Fig. 5b–g, both the surface and the interior of the AS have microporous structures, and the pores on the surface, which can easily form the microstructure, are smaller than those in the reservoir layer, which can well absorb the hydrophobic solvent.

To avoid burning or affecting the accuracy of the AS due to overheating durin the carving process, it is necessary to immerse the stamp in water for 2–3 min prior to carving. Then, the pattern designed by AutoCAD or CorelDraw software can be imported into the laser engraving machine to complete the production of the AS (Fig. 5h). During the laser engraving procedure, the spot size is 38 μm (the beam width is 300 μm), and the speed is 500 mm/s. The current used in the engraving procedure is related to the engraving depth. Generally, 10 mA is used. If the engraving is deeper, 15 mA can be used. The specific operation is shown in Fig. 5h.

### Fabrication of μPADs

**Fig. 6:**
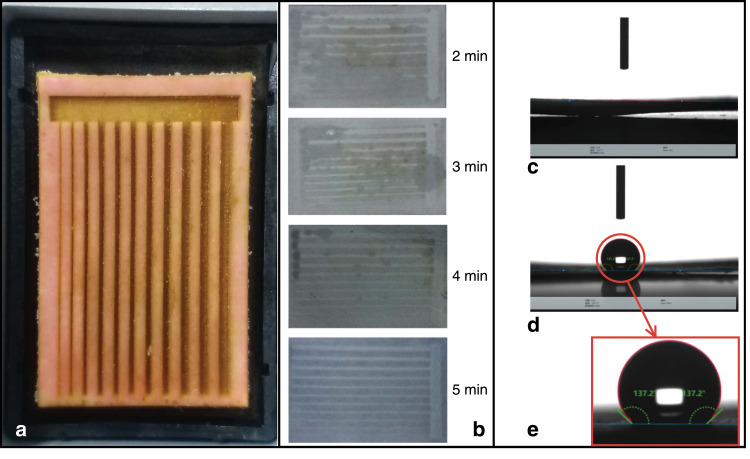
μPAD fabricated by an ASP method. **a** AS with an engraved pattern. **b** ASs with different immersion times. **c** Contact angle between water and the hydrophilic area. **d**, **e** Contact angle between water and the hydrophobic area.

The AS (Fig. 6a), which was engraved, was immersed in the prepared PDMS solvent (PDMS and curing agent mixed in a ratio of 10:1 and put into a vacuum drying box to remove bubbles, which takes ~10 min) to fully absorb the hydrophobic solution (see step 3 in Fig. 5h). By varying the immersion time from 2 to 5 min, as shown in Fig. 6b, we observed that clear patterns can optimally be printed on paper after 5 min of immersion. As shown in Fig. 6c–e, the filter paper treated with PDMS can achieve hydrophobicity. The contact angle between water and the hydrophobic surface can reach 137° (Fig. 6d, e) without affecting the fluid flow in the hydrophilic regions (a contact angle >90° is regarded as hydrophobic; a contact angle <90° is considered hydrophilic).

### Fabrication of the colorimetric card

**Fig. 7:**
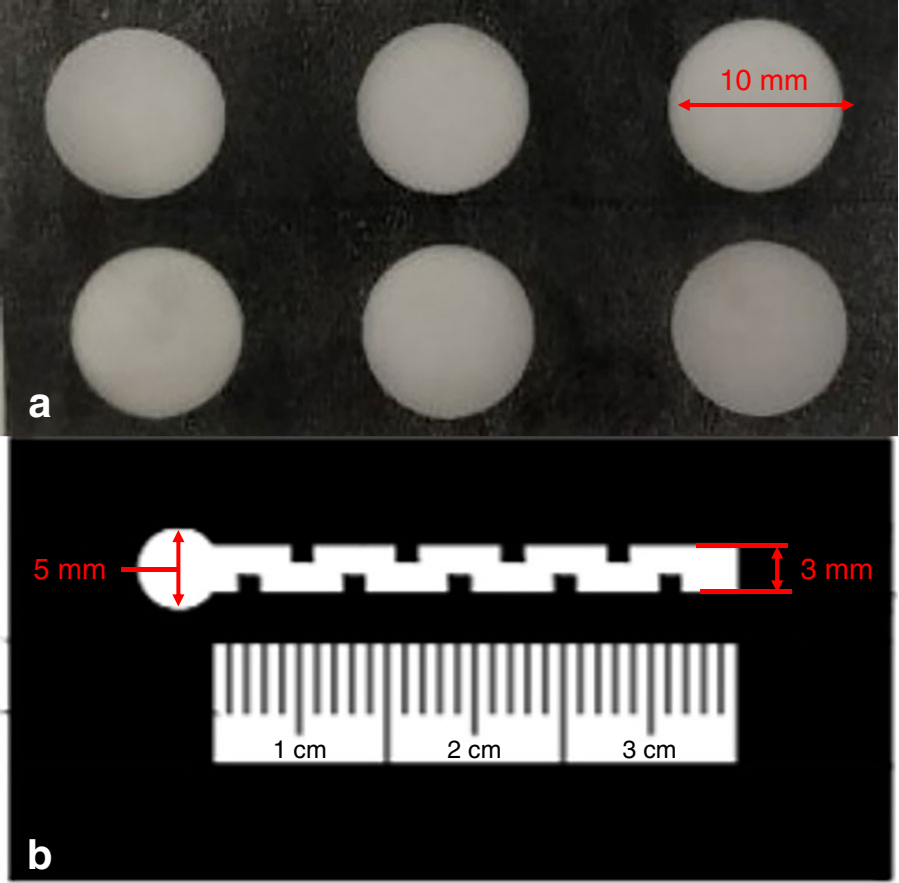
Detection of Cu^2+^ by μPADs. **a** Detection with a colorimetric card. **b** Detection by the distance-based method on a μPAD.

The colorimetric μPADs made with the ASP method (shown in Fig. 7a with a diameter of 10 mm) were immersed in a copper reagent (sodium diethyldithiocarbamate, DDTC) in advance and then dried at room temperature for detection. The principle of making the colorimetric card is that Cu^2+^ reacts with DDTC to produce a brown–yellow complex under weakly alkaline conditions.

The distance-based detection μPAD also needed to be immersed in DDTC solution in advance and dried, and then different concentrations of Cu^2+^ solution were added to the device dropwise (the channel shape was an S-shape, the total length was 30 mm, the width was 3 mm, and there was a liquid storage area with a diameter of 5 mm at the entrance, as shown in Fig. 7b).

### Heavy metal ion extraction device in soil

#### Fabrication of the 3D μPAD

**Fig. 8:**
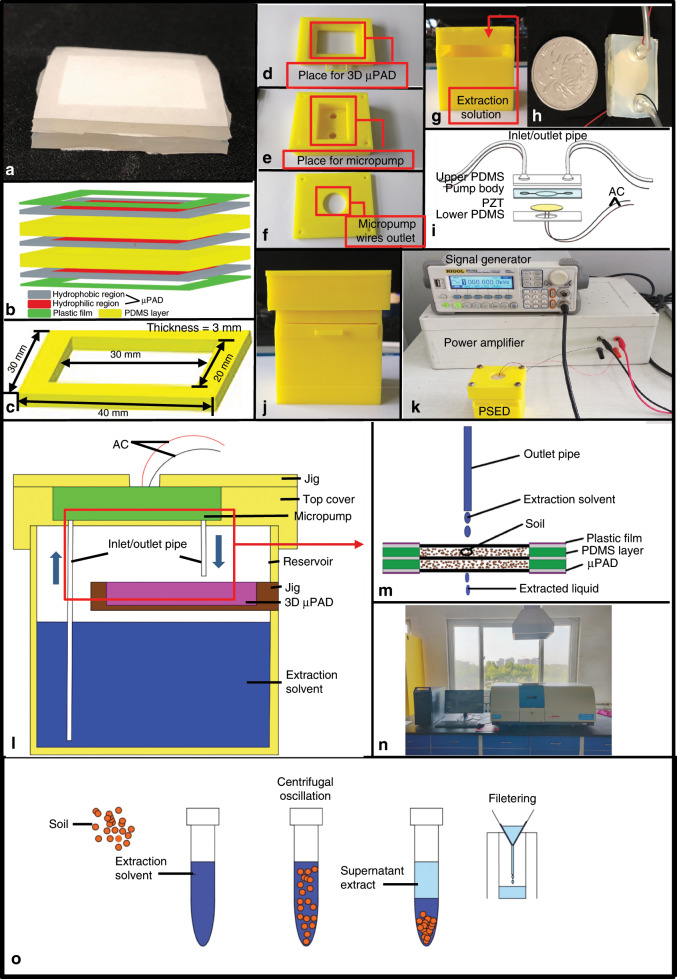
Picture of an integrated soil–liquid extraction device. **a** Physical picture of the 3D μPAD. **b** Structural picture of the 3D μPAD. **c** Size of the PDMS layer. **d** Jig of the μPAD. **e** Top cover. **f** Jig of the micropump. **g** Reservoir. **h** Physical picture of the micropump. **i** Composition of the micropump. **j** Assembled PSED. **k** Experimental platform of the PSED. **l** The inner structure of the PSED. **m** Experimental principle of the PSED. **n** Fire atomic absorption spectrometer (FASS). **o** Experimental principle of traditional soil–liquid extraction.

The principle of making 3D μPADs was to superimpose 2D μPADs to create hydrophilic channels that can flow vertically. The traditional methods of making 3D μPADs include origami^46^, double-sided gluing^47,48^, and stapler binding^49^. Based on the vertical flow characteristics of 3D μPADs, we designed a “3 + 2” structure for a 3D extraction μPAD (Fig. 8a–c). To prevent leakage and loosening during the experiment, three layers of μPADs and two layers of PDMS films were packaged with plastic films, and glue was used to connect the layers. The sizes of the μPAD and PDMS films are shown in Fig. 8c. The hydrophilic region of the μPAD was the same as the hollow part of the PDMS film (30 × 20 mm^2^), and the remaining part was the hydrophobic region. The thickness of the PDMS layer used to store soil or other samples was 3 mm. Furthermore, the 3D μPAD was roughly divided into two storage layers, each of which could hold 1 g of soil.

#### Fabrication of the integrated PSED

The PSED was divided into three main parts: the extraction and filtration section (Fig. 8a–c), the supporting frame (Fig. 8d–g), and the circulating liquid supply section (Fig. 8h–i). As shown in Fig. 8d–g, the four components of the supporting frame, the jig of the μPAD, the top cover, the jig of the micropump, and the reservoir, were all made of PLA and processed by an FDM 3D printer. The fabricated 3D μPAD was placed in the jig (Fig. 8d) and inserted into the reservoir (Fig. 8g) that had been filled with extraction solution. Then, the homemade micropump (see Fig. 8h–i) was put into a groove in the top cover (Fig. 8e) to realize control of the liquid supply in the whole device. To prevent leakage of the micropump during the experiment, a jig (Fig. 8f) was used to fix the micropump, ensuring that the operation and performance of the micropump were not affected during the fixing process, and PZT (lead zirconate titanate) wires were drawn from the hole as shown in Fig. 8f. The size of the reservoir that we designed was 50 × 40 × 60 mm^3^, and the opening of the jig of the μPAD was 45 mm above the bottom; thus, it could store 40 mL of extraction solution (the size of the device can be improved according to different needs). The size of the jig of the μPAD was 40 × 30 mm^2^, and there were 10 mm holes in the wall of the reservoir so that the inlet pipe of the micropump could pass through the gap to the extraction solution.

Finally, the whole device was connected in the way shown in Fig. 8j–k to form a working PSED experimental platform. The inner structure of the PSED after packaging is shown in Fig. 8l. Since a homemade micropump was used in this experiment, a homemade power amplifier was also used for power amplification. Note that if a commercial micropump was used, only the signal generator would be needed.

The principle of extracting heavy metal ions from soil with the PSED is shown in Fig. 8m. The extraction solvent was extracted from the inlet pipe of the micropump and then added dropwise to the 3D μPAD through the outlet pipe. Afterwards, the extracted liquid was infiltrated through the paper and soil by the subsequently added extraction solvent to achieve liquid circulation. The whole extraction area can be automatically wetted due to the hydrophilicity of the paper, ensuring full contact between the extraction solvent and soil. Finally, the extracted liquid was poured out, and the heavy metal ions were detected by atomic absorption spectrometry (see Fig. 8n). The experimental results were compared with those obtained with the traditional centrifugal oscillation and filtration extraction methods (Fig. 8o).

## Supplementary information

Data Set 1

## Data Availability

The authors declare that the data supporting the findings of this study are available within the paper.
